# Evaluating glucose variability through OGTT in early pregnancy and its association with hypertensive disorders of pregnancy in non-diabetic pregnancies: a large-scale multi-center retrospective study

**DOI:** 10.1186/s13098-023-01103-z

**Published:** 2023-06-09

**Authors:** Sho Tano, Tomomi Kotani, Takafumi Ushida, Masato Yoshihara, Kenji Imai, Noriyuki Nakamura, Yukako Iitani, Yoshinori Moriyama, Ryo Emoto, Sawako Kato, Shigeru Yoshida, Mamoru Yamashita, Yasuyuki Kishigami, Hidenori Oguchi, Shigeyuki Matsui, Hiroaki Kajiyama

**Affiliations:** 1 Department of Obstetrics and Gynecology, Nagoya University Graduate School of Medicine, Nagoya, Aichi Japan; 2 Department of Obstetrics, Perinatal Medical Center, TOYOTA Memorial Hospital, Nagoya, Aichi Japan; 3 Division of Perinatology, Center for Maternal-Neonatal Care, Nagoya University Hospital, Nagoya, Achi Japan; 4 Department of Obstetrics and Gynecology, Fujita Health University School of Medicine, Nagoya, Aichi Japan; 5 Department of Biostatistics, Nagoya University Hospital, Nagoya, Aichi Japan; 6 Department of Nephrology, Nagoya University Graduate School of Medicine, Nagoya, Aichi Japan; 7 Kishokai Medical Corporation, Inazawa, Aichi Japan

**Keywords:** Glucose variability, Hypertensive disorders of pregnancy, OGTT

## Abstract

**Background:**

Recent evidence suggests increased glucose variability (GV) causes endothelial dysfunction, a central pathology of hypertensive disorders of pregnancy (HDP). We aimed to investigate the association between GV in early pregnancy and subsequent HDP development among non-diabetes mellitus (DM) pregnancies.

**Methods:**

This multicenter retrospective study used data from singleton pregnancies between 2009 and 2019. Among individuals who had 75 g-OGTT before 20 weeks of gestation, we evaluated GV by 75 g-OGTT parameters and examined its relationship with HDP development, defining an initial-increase from fasting-plasma glucose (PG) to 1-h-PG and subsequent-decrease from 1-h-PG to 2-h-PG.

**Results:**

Approximately 3.0% pregnancies (802/26,995) had 75 g-OGTT before 20 weeks of gestation, and they had a higher prevalence of HDP (14.3% vs. 7.5%). The initial-increase was significantly associated with overall HDP (aOR 1.20, 95% CI 1.02–1.42), and the subsequent-decrease was associated with decreased and increased development of early-onset (EoHDP: aOR 0.56, 95% CI 0.38–0.82) and late-onset HDP (LoHDP: aOR 1.38, 95% CI 1.11–1.73), respectively.

**Conclusions:**

A pattern of marked initial-increase and minor subsequent-decrease (i.e., sustained hyperglycemia) was associated with EoHDP. Contrarily, the pattern of marked initial-increase and subsequent-decrease (i.e., increased GV) was associated with LoHDP. This provides a new perspective for future study strategies.

**Supplementary Information:**

The online version contains supplementary material available at 10.1186/s13098-023-01103-z.

## Background

Hypertensive disorders of pregnancy (HDP) are pregnancy-specific disorders with an incidence of 8–10%, a major cause of maternal mortality [1]. The “two-stage model” has been proposed as a representative hypothesis of pathophysiology [2]. Briefly, at stage 1, immunological or other factors cause an insufficient remodeling of spiral arteries, leading to poor placentation occurring until 20 weeks of gestation (weeks of gestation; wks). At stage 2, endothelial dysfunction is caused by imbalances of factors related to stage 1 or maternal predispositions, e.g., obesity, genetics, and nutrition [3, 4]. HDP has two subtypes: early-onset HDP (EoHDP, developed at  < 34 wks) and late-onset HDP (LoHDP, developed at  ≥ 34 wks) [5, 6]. The pathophysiological difference between the two subtypes remains unclear. However, accumulating evidence has suggested that endothelial dysfunction in EoHDP is often associated with poor placentation, while that in most LoHDP is affected by maternal predispositions [2, 5, 7].

There is increasing evidence of the clinical value of glucose variability (GV) [8–13]. Oxidative stress, a key player in endothelial dysfunction [9, 14], is induced by increased GV more effectively than sustained hyperglycemia [15]. Increased GV also reduces endothelial progenitor cells [16], and is more deleterious than sustained hyperglycemia with molecular and cytological changes [12, 17]. Clinical studies have also revealed that increased GV is associated with endothelial dysfunction and cardiovascular disease in diabetes mellitus (DM) patients and healthy individuals [10, 11, 13, 18, 19].

The gold standard for GV assessment is continuous glucose monitoring (CGM) [20]. Accordingly, the association between GV and perinatal prognosis in pregnancies with type 1 DM (T1DM) has been accumulating recently [21, 22]. However, it is challenging to use CGM in pregnant women universally. To our best knowledge, there are no studies about the association between GV and perinatal outcomes in non-DM pregnant women. Recently, it has been reported that oral glucose tolerance tests (OGTT) are adopted for GV assessment when CGM is unavailable [23, 24], allowing GV assessment in non-DM pregnant women.

Since HDP is reported to exacerbate maternal insulin resistance [25], it is necessary to determine the association between GV in early pregnancy before the onset of HDP and subsequent HDP development. The OGTT in early pregnancy is controversial because of the lack of evidence on the evaluation and treatment of gestational diabetes mellitus (GDM) detected in early pregnancy [26–28]. However, GDM diagnosed in early pregnancy has comparable risks to pre-existing DM [29]. This inconsistency was identified as a research gap [30], and there is an urgent need to accumulate evidence. Hence, we investigated the association between GV and the subsequent development of HDP (EoHDP and LoHDP) using OGTT parameters in early pregnancy.

## Methods

### Study design and participants

**Fig. 1:**
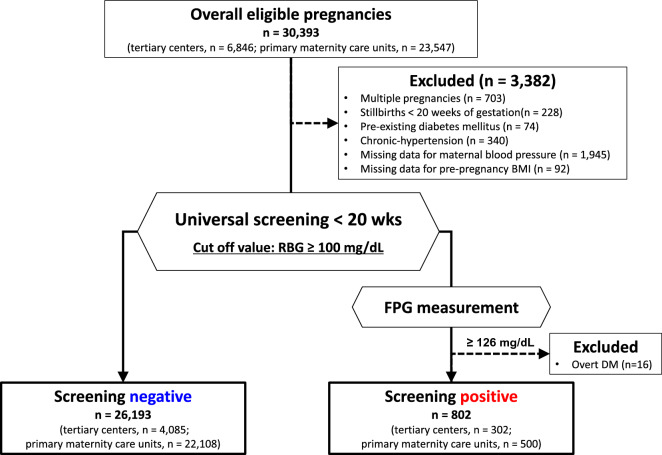
Flow chart of the study participants. Among 30,393 individuals, 26,995 were eligible after excluding 3,398 individuals. *BMI* body mass index, *RBG* random blood glucose levels, *wks* weeks of gestation, *DM* diabetes mellitus, *FPG* fasting plasma glucose level

This multicenter retrospective study used electronic data obtained from 2 tertiary centers (Nagoya University Hospital and TOYOTA Memorial Hospital, Aichi, Japan) and 12 private maternity facilities (Kishokai Medical Corporation, Aichi, Japan). Data were collected for women aged  ≥ 15 years who gave birth in 2009–2019 and whose data before 20 wks were available. Women with multiple pregnancies, stillbirth before 20 wks, chronic hypertension, pre-existing DM, overt-DM, and data missing for blood pressure and pre-pregnant body mass index (BMI) were excluded (Fig. 1). Although chronic hypertension is one of the subtypes of HDP, this group was excluded to evaluate the association between GV and HDP development.

### Study outcomes

The primary outcome is developing HDP (EoHDP or LoHDP) in non-DM pregnancies. In this study, we analyzed the association between the parameters of the 75 g-OGTT performed before 20 wks and the subsequent HDP development.

### Diagnosis of overt-DM/GDM

Pregnant women were screened for random blood glucose (RBG) around 12–14 wks, according to the guideline with setting cut-off value at RBG of  ≥ 100 mg/dL (5.6 mmol/L) [31, 32]. For screening-positive patients, fasting plasma glucose level (FPG) was measured, and patients with FPG of  ≥ 126 mg/dL (7.0 mmol/L) were diagnosed as overt-DM. For non-overt-DM individuals, 75 g-OGTT was conducted with cutoff values of  ≥ 92 mg/dL (5.1 mmol/L) for FPG,  ≥ 180 mg/dL (10 mmol/L) for 1 h plasma glucose level after loading (1-h-PG), and ≥ 153 mg/dL (8.5 mmol/L) for 2 h plasma glucose level after loading (2-h-PG). GDM was diagnosed if at least one of the three aforementioned glycemic levels was above the threshold. After the 1st screening of negative individuals, the 2nd screening was conducted at around 24–28 wks using RBG with the same cutoff value or a 50 g-glucose challenge test with a cutoff value ≥ 140 mg/dL (7.8 mmol/L) for 1-h-PG was performed, followed by 75 g-OGTT with the same cutoff value. In Japan, oral diabetes medications are contraindicated for pregnant women, so treatment was by diet and insulin injections.

### Definitions

HDP was defined as the development of new hypertension (systolic blood pressure of  ≥ 140 mmHg or diastolic blood pressure of  ≥ 90 mmHg) after 20 wks [33]. Self-reported maternal height and body weight were used to calculate pre-pregnant BMI (kg/m^2^). The participants were categorized as underweight (< 18.5 kg/m^2^), normal-weight (≥ 18.5 to  < 25.0 kg/m^2^), and overweight (≥ 25.0 kg/m^2^) [4]. Assisted reproductive technology (ART) was defined as in vitro fertilization or intracytoplasmic sperm injection. Large-for-gestational age (LGA) and small-for-gestational age (SGA) infants were diagnosed using the Japanese standards [34].

**Fig. 2:**
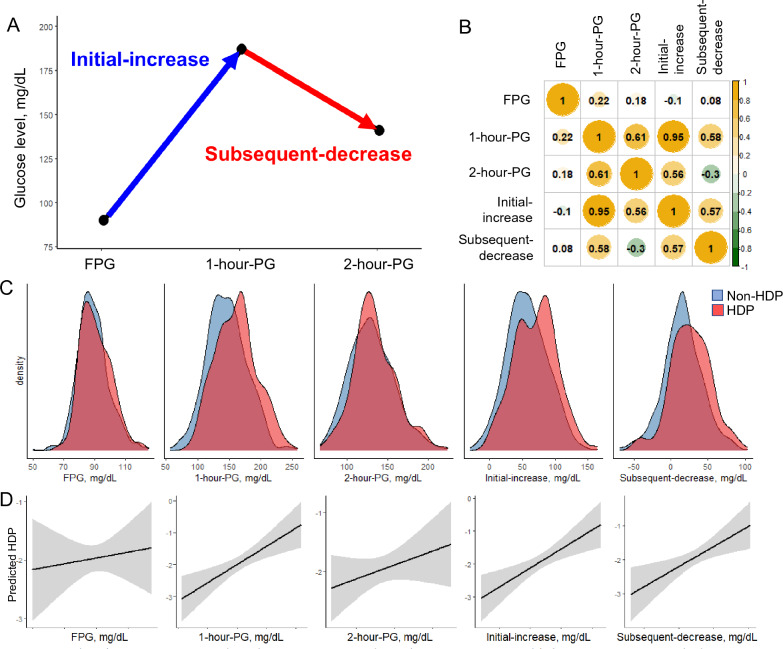
Association between 75 g-OGTT parameters and HDP (n = 802). **A** Initial-increase was defined as an increase in FPG to 1 h plasma glucose level after loading (1-h-PG). Subsequent-decrease was defined as a decrease in 1-h-PG to 2 h plasma glucose level after loading (2-h-PG). **B** The scale on the right side and values inside the columns of the correlogram indicate the Pearson correlation value. **C** The X-axis of the density plot represents the value of 75 g-OGTT parameters. Blue and red parts represent the non-HDP and HDP group, respectively. **D** The fitted value (solid-line) and 95% CI (shaded-area) of HDP for each 75 g-OGTT parameter were calculated by a generalized additive model using maternal age, pre-pregnant BMI, and primiparity as covariables. *HDP* hypertensive disorders of pregnancy, *95% CI* 95% confidence interval, *BMI* pre-pregnant body mass index, *FPG* fasting plasma glucose level

While assessing GV using OGTT, 1-h-PG and incremental glucose peak (IGP), defined as the increment from FPG to peak value, are equivalent to the GV in CGM [23]. We also analyzed other parameters such as the increase from FPG to 1-h-PG as initial-increase, and the decrease from 1-h-PG to 2-h-PG as subsequent-decrease to evaluate GV from multiple perspectives (Fig. 2A).

### Statistical analysis

Clinical characteristics are presented as mean ± standard deviation for continuous variables and number (%) for categorical variables. Baseline characteristics were evaluated by applying standardized mean difference to avoid identifying spurious statistical associations in the large data-set. Standardized mean differences of  ≥ 0.1 were considered indicative of imbalance. We also applied a propensity score (PS) matched analysis. We calculated the PS for screening-positive using a logistic regression model that included the following variables collected before the universal screening: maternal age, pre-pregnant BMI, primiparity, and ART pregnancy. One-to-one nearest-neighbor matching without a replacement was performed for the estimated propensity scores of the patients using a caliper width set at 20% of the standard deviation for the PS. Crude odds ratios (cORs) and adjusted ORs (aORs) of HDP for 75 g-OGTT parameters were calculated by univariable and multivariable logistic regression analyses using known HDP risk factors: maternal age, pre-pregnant BMI, current smoker status, primiparity, and ART as covariables [4, 33]. Correlation coefficients (r) of 75 g-OGTT parameters were calculated based on Pearson's test. The non-linear association between each 75 g-OGTT parameter and HDP was evaluated using generalized additive models, adjusting for known HDP risk factors [4, 33]. Statistical analyses were conducted using SPSS, version 28.0 (IBM Corp.) and R, version 4.1.3 (https://cran.r-project.org/).

## Results

### Participants

Among 30,393 pregnant women (tertiary centers, n = 6,846; primary maternity care units, n = 23,547, Fig. 1), 3,398 women were excluded because of multiple pregnancies (n = 703), stillbirth before 20 weeks (n = 228), pre-existing DM (n = 74), chronic hypertension (n = 340), missing data for maternal blood pressure (n = 1,945) and pre-pregnant BMI (n = 92), and overt DM (n = 16). Finally, 26,995 women were included.

### Background of the screening-positive group

Approximately 3.0% (802/26,995) of individuals was screening-positive. As shown in Table 1, before matching, maternal age (32.9 ± 5.1 vs. 31.5 ± 4.9 years), pre-pregnant BMI (22.5 ± 4.3 vs. 20.9 ± 3.1 kg/m^2^), and the incidence of GDM (54.0% vs. 2.8%) were significantly higher in the screening-positive group than in the screening-negative group. HDP was also significantly more frequent in the screening-positive group than in the screening-negative group (14.3% vs. 7.5%). Regarding the HDP subtypes, the incidence of LoHDP was significantly higher in the screening-positive group than in the screening-negative group (11.2% vs. 4.8%), but the difference in the prevalence of EoHDP was not significant (3.1% vs. 2.7%). There were 798 (≅ 99.5%) patients in the screening-positive group matched to those in the screening-negative group, and the covariates of maternal age, pre-pregnant BMI, primiparity, and ART pregnancy, were balanced between groups after matching (standardized mean difference < 0.1). The incidences of GDM (53.9% vs. 3.3%) and HDP (14.3% vs. 9.3%) were significantly higher in the matched screening-positive group (standardized mean difference ≥ 0.1) .

**Table 1:** Baseline characteristics and perinatal outcomes

	Overall cohort		Standardized mean difference^¶^	PS matched cohort		Standardized mean difference^¶^
	screening positive	Screening negative		Screening positive	Screening negative	
	n = 802	n = 26,193		n = 798	n = 798	
Maternal age, years	32.9 ± 5.1	31.5 ± 4.9	**0.27**	32.9 ± 5.1	33.0 ± 4.9	0.02
Pre-pregnant BMI, kg/m^2^	22.5 ± 4.3	20.9 ± 3.1	**0.37**	22.5 ± 4.3	22.3 ± 3.8	0.05
Overweight	163 (20.3)	2,207 (8.4)	**0.40**	159 (19.9)	151 (18.9)	0.03
Underweight	90 (11.2)	4,615 (17.6)	**0.17**	90 (11.3)	78 (9.8)	0.05
Primiparity	355 (44.3)	11,707 (44.7)	0.01	355 (45.5)	340 (42.6)	0.06
ART	108 (13.5)	1,584 (6.0)	**0.30**	107 (13.4)	107 (13.4)	0.00
Current smoker	10 (2.1)	322 (1.2)	0.07	10 (2.1)	9 (1.4)	0.05
GDM	433 (54.0)	730 (2.8)	**2.34**	430 (53.9)	23 (3.3)	**1.10**
GA at the 75 g-OGTT, weeks	13.5 ± 1.7			13.5 ± 1.7		
FPG, mg/dL	89.2 ± 10.0			89.2 ± 10.0		
1-h-PG, mg/dL	148.6 ± 31.9			148.5 ± 31.9		
2-h-PG, mg/dL	131.3 ± 27.2			131.1 ± 27.2		
Initial-increase, mg/dL	59.3 ± 31.3			59.3 ± 31.3		
Subsequent-decrease, mg/dL	17.3 ± 26.6			17.3 ± 26.6		
HDP	115 (14.3)	1,968 (7.5)	**0.25**	114 (14.3)	74 (9.3)	**0.16**
EoHDP	25 (3.1)	700 (2.7)	0.02	24 (3.0)	31 (3.9)	0.05
LoHDP	90 (11.2)	1,267 (4.8)	**0.28**	90 (11.3)	42 (5.3)	**0.22**
Stillbirth ≥ 20 weeks	4 (0.5)	146 (0.6)	0.01	4 (0.5)	5 (0.6)	0.01
GA at delivery, weeks	39.3 ± 1.7	39.3 ± 1.9	0.00 00	39.3 ± 1.7	39.2 ± 2.1	0.06
Neonatal sex, male	409 (51.0)	12,118 (46.3)	0.09	405 (51.3)	402 (52.3)	0.02
Birthweight, g	3,084 ± 459	3,028 ± 453	**0.12**	3,084 ± 459	3,032 ± 490	**0.11**
Large for gestational age	126 (15.7)	2,722 (10.4)	**0.17**	125 (15.7)	117 (14.8)	0.03
Small for gestational age	50 (6.2)	1,795 (6.9)	0.03	50 (6.3)	48 (6.1)	0.01
Placental weight, g	592.5 ± 114.3	584.2 ± 114.7	0.07	592.2 ± 114.4	592.4 ± 113.5	0.00

Bold values represent statistically significant

Continuous variables were represented as mean ± standard division. Categorical variables were represented as n (%)

^¶^Differences of ≥ 0.1 represent meaningful differences in covariates between groups (Bold)

*PS* propensity score, *RBG* random blood glucose levels, *BMI* body mass index, *ART* assisted reproductive technology, *GDM* gestational diabetes mellitus, *GA* gestational age, *wks* weeks of gestation, *OGTT* oral glucose challenge test, *HDP* hypertensive disorders of pregnancy, *EoHDP* early-onset HDP, *LoHDP* late-onset HDP

### 75 g-OGTT parameters evaluation

We analyzed 75 g-OGTT parameters among individuals with screening-positive (n = 802). Mean FPG, 1-h-PG, and 2-h-PG were 89.2 ± 10.0, 148.6 ± 31.9, and 131.3 ± 27.2 mg/dL, respectively (Table 1). Figure 2B demonstrates a very strong correlation between initial-increase and 1-h-PG (r = 0.95), but the correlation between initial-increase and subsequent-decrease was moderate (r = 0.57). The correlation between subsequent-decrease and 1-h-PG was also moderate (r = 0.58). In contrast, the correlations between FPG and other parameters were very weak (−0.1 ≤ r ≤ 0.22).

The distributions of 75 g-OGTT parameters were compared between non-HDP (blue) and HDP (red) groups using the density plot (Fig. 2C). Although FPG and 2-h-PG showed almost similar distribution patterns in the two groups, initial-increase changed dramatically and showed two peaks in the HDP group: one located near the peak in the non-HDP group and another located at a higher value. The 1-h-PG and subsequent-decrease distribution showed a right shift of the peak in the HDP group. The 1-h-PG, initial-increase, and subsequent-decrease were linearly positively associated with HDP development (Fig. 2D).

### GV was associated with HDP

These values were also compared between the non-HDP group (blue) and the two HDP subgroups, EoHDP (yellow) and LoHDP (red), using a density plot (Additional file 1: Figure S1A). The parameters of 1-h-PG and initial-increase were positively associated with both subtypes (Additional file 1: Figure S1B–C). Contrastingly, the distribution of subsequent-decrease in the LoHDP group was right-shifted, whereas that in the EoHDP was a left-shifted, bimodal peak (Additional file 1: Figure S1A). Accordingly, subsequent-decrease was negatively and positively associated with EoHDP (Additional file 1: Figure S1B) and LoHDP (Additional file 1: Figure S1C), respectively. Similarly, the distribution of 2-h-PG was right-shifted in the EoHDP group compared to the other groups (Additional file 1: Figure S1A) and had a linear positive association with EoHDP (Additional file 1: Figure S1B). However, there was no positive association with LoHDP (Additional file 1: Figure S1C). For FPG, the association was nonlinear with both EoHDP and LoHDP (Additional file 1: Figure S1B–C).

As shown in Table 2, subsequent-decrease was negatively associated with EoHDP (aOR 0.56, 95% CI 0.38–0.82, per 20-mg/dL increment) and positively associated with LoHDP (aOR 1.38, 95% CI 1.11–1.73, per 20-mg/dL increment). The aOR of LoHDP for subsequent-decrease was reproduced in the sensitivity analyses (Additional file 2: Table S1): 1.45 (95% CI 1.21–1.74, Model 1) and 1.46 (95% CI 1.21–1.75, Model 2), respectively. The aOR of EoHDP for subsequent-decrease showed a similar trend in the sensitivity analyses (Additional file 2: Table S1), but these values were not significant (aOR 0.78, 95% CI 0.57–1.07, Model 1; aOR 0.77, 95% CI 0.56–1.05, Model 2). For initial-increase (Table 2), it was significantly associated with overall HDP and EoHDP with aORs of 1.20 (95%CI 1.02–1.42) and 1.57 (95% CI 1.15–2.15) per 20-mg/dL increment, respectively, but the association with LoHDP was not significant: aOR of 1.08 (95% CI 0.90–1.30). However, the aOR of LoHDP for initial-increase was significant in the sensitivity analyses: aOR of 1.25 (95% CI 1.08–1.46) (Models 3–4, Additional file 2: Table S1). The 1-h-PG and IGP were significantly associated with HDP and LoHDP: aORs of HDP for each were 1.26 (95% CI 1.10–1.44) and 1.25 (95% CI 1.08–1.45) per 20-mg/dL increment, respectively; and aORs of LoHDP for each were 1.24 (95% CI 1.07–1.43) and 1.21 (95% CI 1.03–1.41) per 20-mg/dL increment, respectively (Models 5–6, Additional file 2: Table S1). Those parameters were not significantly associated with EoHDP.

**Table 2:** 75 g-OGTT parameters and HDP in individuals with RBG of ≥ 100 mg/dL (n = 802)

	HDP (n = 115)		EoHDP (n = 25)		LoHDP (n = 90)	
	cOR (95%CI)	aOR^§^ (95%CI)	cOR (95%CI)	aOR^§^ (95%CI)	cOR (95%CI)	aOR^§^ (95%CI)
Age, years	1.02 (0.98–1.06)	1.00 (0.96–1.05)	0.98 (0.90–1.05)	0.96 (0.88–1.05)	1.02 (0.98–1.07)	1.02 (0.97–1.07)
Pre-pregnant BMI, kg/m^2^	1.16* (1.11–1.21)	1.16* (1.11–1.21)	1.15* (1.08–1.23)	1.17* (1.08–1.26)	1.13* (1.09–1.18)	1.13* (1.08–1.19)
Primiparity	1.51* (1.02–2.24)	1.58* (1.02–2.46)	0.99 (0.44–2.21)	0.99 (0.42–2.32)	1.67* (1.07–2.59)	1.72* (1.06–2.78)
ART	1.77* (1.06–2.94)	1.99* (1.10–3.58)	1.23 (0.41–3.66)	1.95 (0.57–6.72)	1.87* (1.08–3.25)	1.88 (1.00–3.54)
FPG^†^	1.48* (1.01–2.18)	1.13 (0.73–1.75)	2.10* (1.00–4.40)	1.82 (0.81–4.08)	1.29 (0.84–1.98)	0.92 (0.56–1.51)
Initial-increase^†^	1.29* (1.14–1.47)	1.20* (1.02–1.42)	1.22 (1.00–1.57)	1.57* (1.15–2.15)	1.29* (1.12–1.48)	1.08 (0.90–1.30)
Subsequent-decrease^†^	1.36* (1.17–1.58)	1.12 (0.92–1.37)	0.88 (0.65–1.18)	0.56* (0.38–0.82)	1.52* (1.28–1.81)	1.38* (1.11–1.73)

Bold values represent statistically significant

*RBG* random blood glucose levels, *HDP* hypertensive disorders of pregnancy, *EoHDP* early-onset HDP, *LoHDP* late-onset HDP, *cOR* crude odds ratio, *aOR* adjusted odds ratio, *95% CI* 95% confidence interval, *BMI* body mass index, *ART* assisted reproductive technology, *FPG* fasting plasma glucose level

^§^aORs were adjusted for maternal age, pre-pregnant BMI, primiparity, ART, FPG, initial-increase, and subsequent-decrease

^†^per 20-mg/dL increment

^*^Statistically significant

**Fig. 3:**
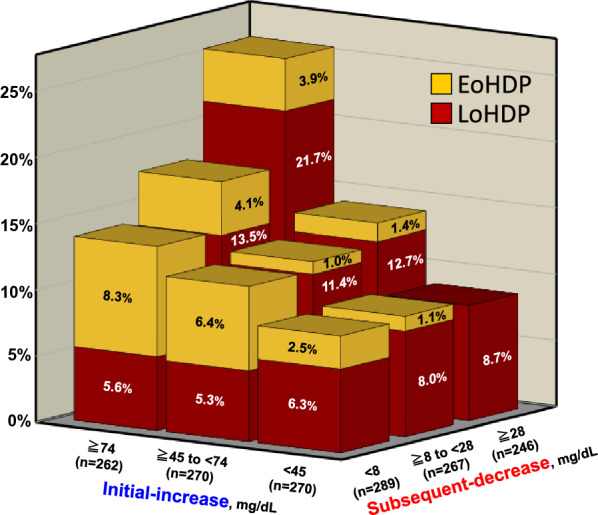
Three-dimensional bar chart representing the frequency of EoHDP and LoHDP (n = 802). The frequencies of EoHDP (yellow-cuboid) and LoHDP (red-cuboid) are represented on the Z axis, with the classified initial-increase and subsequent-decrease forming the axes of X and Y, respectively. *EoHDP* early-onset hypertensive disorders of pregnancy, *LoHDP* late-onset hypertensive disorders of pregnancy

The three-dimensional bar chart represents the frequency of HDP on the Z-axis, and the X and Y axes are formed with groups for trisected initial-increase and subsequent-decrease, respectively (Fig. 3). The HDP frequency tended to increase with increasing values of initial-increase and subsequent-decrease, except in the row with an initial-increase of < 45 mg/dL. The group with both the largest initial-increase (≥ 74 mg/dL) and subsequent-decrease (≥ 28 mg/dL) had the highest frequency of HDP (25.7%), the majority of which was LoHDP (red-cuboid, 21.7%). Contrarily, the group with the largest initial-increase (≥ 74 mg/dL) and the smallest subsequent-decrease (< 8 mg/dL) had the highest frequency of EoHDP (yellow-cuboid, 8.3%).

### GV was associated with HDP also in non-GDM individuals

Approximately 12.7% (47/369) developed HDP, EoHDP (n = 9) and LoHDP (n = 38), among the individuals who did not meet the GDM diagnostic criteria (non-GDM, Additional file 3: Table S2). Initial-increase was significantly associated with HDP: aOR of 1.52 (95% CI 1.03–2.26). Subsequent-decrease was not significantly associated with overall HDP after adjustment (aOR 1.20, 95% CI 0.80–1.78). However, subsequent-decrease was significantly associated with EoHDP and LoHDP with aORs of 0.35 (95% CI 0.13–0.94) and 1.54 (95% CI 1.01–2.36), respectively. After dividing participants into three groups with subsequent-decrease (< 8, ≥ 8 to < 28, and ≥ 28 mg/dL), the frequency of LoHDP was the highest in the largest group: 5.4% (7/129), 7.2% (10/138), and 20.6% (21/102), respectively (data not shown).

## Discussion

This study is the first to demonstrate the association between GV (assessed by 75 g-OGTT performed in early pregnancy) and subsequent development of HDP in non-DM individuals. Briefly, the pattern of a marked initial-increase followed by a marked subsequent-decrease (i.e., increased GV) was significantly associated with subsequent LoHDP development. Meanwhile, the pattern of poor or lack of subsequent-decrease after the marked initial-increase (i.e., sustained hyperglycemia) was associated with subsequent EoHDP development. These trends were also observed in non-GDM individuals. These findings suggest that more attention is needed in this group that has been overlooked thus far.

The present study findings suggest that new parameters of 75 g-OGTT such as initial-increase and subsequent-decrease were associated with subsequent HDP development in non-DM individuals in the screening-positive cohort. A new finding was that subsequent-decrease was negatively and positively associated with EoHDP and LoHDP development, respectively. Poor placentation, a major etiology of EoHDP development [2, 5, 7], is associated with early exposure to sustained hyperglycemia [29, 35–40]. Endothelial dysfunction in LoHDP development is not commonly accompanied by poor placentation [2, 5], and it is reported to be induced by increased GV rather than sustained hyperglycemia [8, 41]. The present study’s results are consistent with these findings. Since subsequent-decrease was negatively associated with EoHDP but positively associated with LoHDP, the association between subsequent-decrease and overall HDP development was not significant. These associations were also confirmed in non-GDM individuals, reinforcing the involvement of GV in the pathogenesis of LoHDP. IGP and 1-h-PG, reported indicators of GV [23], were also shown to be associated with HDP and LoHDP development, but not with EoHDP development. The association between 1-h-PG at OGTT performed after 20 wks and HDP has been reported previously [42–45], and that between increased GV and HDP was also reported in T1DM patients [46]. However, this study is the first to demonstrate the association between GV in early pregnancy and HDP in non-DM individuals. Although FPG is also associated with HDP [42–44], the association was not detected herein. This discrepancy might be due to the study populations involved; participants were limited to those with screening-positive. Hence, the population with a sufficiently low level of FPG would have been small.

The findings of this study suggest that by assessing GV with OGTT at low cost, women at high risk of developing HDP, which typically occurs after 20 wks, can be identified before 20 wks. It has a significant practical value in the clinical setting, including early intervention [47]. However, early pregnancy is also a period when morning sickness is likely to occur. Thus, performing OGTT, which requires fasting and drinking a glucose solution, can be stressful for women. Therefore, it is important that selective OGTT be performed on the minimum number of pregnant women who need it.

### Strengths and limitations

The strength of this study is that the participants were selected based on universal screening with a single criteria routinely used at both tertiary care centers and primary maternity care units. This strategy minimized selection bias.

This study has several limitations. First, insulin resistance was not examined. There was also no data on pre-pregnancy insulin resistance. However, women who had pre-pregnancy insulin resistance corresponding to diabetes were excluded from the analyses according to the exclusion criteria. Individuals with high insulin resistance have a delayed time to glucose peak [48–50]. In this study, the group with a marked initial-increase and poor or lack of subsequent-decrease, suggesting delayed time to glucose peak, had the highest frequency of EoHDP. This finding was consistent with those of previous reports about the association between impaired insulin sensitivity and EoHDP [51]. Second, the incidence of HDP was low, especially in non-GDM individuals. The values of 95% CI were wide in multivariable analyses; however, the differences in trends between LoHDP and EoHDP were shown, and the results were consistent in all subgroup analyses. Third, it was impossible to stratify the results with or without obesity due to the small number of overweight women. Instead, multivariable analyses were conducted using pre-pregnant BMI as a covariant to account for pre-pregnancy overweight. Fourth, 75 g-OGTT was performed only in individuals with screening-positive. Thus, this association in individuals with screening-negative remains unknown. Nevertheless, this study demonstrated a lower frequency of HDP in individuals with screening-negative. Thus, GV screening seems meaningless for individuals with screening-negative.

## Conclusions

This study revealed that GV in early pregnancy assessed by 75 g-OGTT performed in non-DM individuals with screening-positive based on RBG of  ≥ 100 mg/dL was associated with HDP. LoHDP and EoHDP were associated with increased GV and sustained hyperglycemia, respectively. These findings provide a new perspective on HDP risk stratification and the basis for future studies to reduce HDP risk by optimizing GV during pregnancy, or to better understand the differences in pathogenesis between EoHDP and LoHDP.

## Supplementary Information

**Additional file 1: Figure S1.** Association between 75 g-OGTT parameters and HDP subtypes (n = 802). **A** The X-axis of the density plot represents the 75 g-OGTT parameters. Blue, yellow, and red parts represent the non-HDP, EoHDP, and LoHDP group, respectively. **B** The fitted value (solid lines) and 95% CI (shaded areas) of EoHDP for each 75 g-OGTT parameters were calculated by a generalized additive model using maternal age, pre-pregnant BMI, and primiparity as covariables. **C** The fitted value (solid lines) and 95% CI (shaded areas) of LoHDP for each 75 g-OGTT parameter were calculated by a generalized additive model using maternal age, pre-pregnant BMI, and primiparity as covariables. FPG, fasting plasma glucose level; PG, plasma glucose level; HDP, hypertensive disorders of pregnancy; EoHDP, early-onset HDP; LoHDP, late-onset HDP; BMI, pre-pregnant body mass index.

**Additional file 2: Table S1.** The association between the 75 g-OGTT parameters and HDP (n = 802).

**Additional file 3: Table S2.** 75 g-OGTT parameters and HDP in non-GDM individuals (n = 369).

## Data Availability

The raw data supporting the conclusions of this article are available upon reasonable request, and with the permission of TOYOTA Memorial Hospital and Kishokai Medical Corporation.

## Abbreviations

EoHDP: Ealy-onset hypertensive disorders of pregnancy
FPG: Fasting plasma glucose level
GV: Glucose variability
HDP: Hypertensive disorders of pregnancy
LoHDP: Late-onset HDP
PG: Plasma glucose level
RBG: Random blood glucose level
wks: Weeks of gestation

## References

[1] Garovic VD, Dechend R, Easterling T, Karumanchi SA, McMurtry Baird S, Magee LA, Rana S, Vermunt JV, August P, American Heart Association Council on H (2022). Hypertension in pregnancy: diagnosis, blood pressure goals, and pharmacotherapy: a scientific statement from the American Heart Association. Hypertension.

[2] Roberts JM, Hubel CA (2009). The two stage model of preeclampsia: variations on the theme. Placenta.

[3] Vikse BE, Irgens LM, Leivestad T, Skjaerven R, Iversen BM (2008). Preeclampsia and the risk of end-stage renal disease. N Engl J Med.

[4] Tano S, Kotani T, Ushida T, Yoshihara M, Imai K, Nakano-Kobayashi T, Moriyama Y, Iitani Y, Kinoshita F, Yoshida S (2021). Annual body mass index gain and risk of hypertensive disorders of pregnancy in a subsequent pregnancy. Sci Rep.

[5] Steegers EA, von Dadelszen P, Duvekot JJ, Pijnenborg R (2010). Pre-eclampsia. Lancet.

[6] Tranquilli AL, Brown MA, Zeeman GG, Dekker G, Sibai BM (2013). The definition of severe and early-onset preeclampsia. Statements from the International Society for the Study of Hypertension in Pregnancy (ISSHP). Pregnancy Hypertens.

[7] Wikstrom AK, Larsson A, Akerud H, Olovsson M (2009). Increased circulating levels of the antiangiogenic factor endostatin in early-onset but not late-onset preeclampsia. Reprod Sci.

[8] Esposito K, Nappo F, Marfella R, Giugliano G, Giugliano F, Ciotola M, Quagliaro L, Ceriello A, Giugliano D (2002). Inflammatory cytokine concentrations are acutely increased by hyperglycemia in humans: role of oxidative stress. Circulation.

[9] Papachristoforou E, Lambadiari V, Maratou E, Makrilakis K (2020). Association of glycemic indices (hyperglycemia, glucose variability, and hypoglycemia) with oxidative stress and diabetic complications. J Diabetes Res.

[10] Ceriello A, Esposito K, Piconi L, Ihnat MA, Thorpe JE, Testa R, Boemi M, Giugliano D (2008). Oscillating glucose is more deleterious to endothelial function and oxidative stress than mean glucose in normal and type 2 diabetic patients. Diabetes.

[11] Di Flaviani A, Picconi F, Di Stefano P, Giordani I, Malandrucco I, Maggio P, Palazzo P, Sgreccia F, Peraldo C, Farina F (2011). Impact of glycemic and blood pressure variability on surrogate measures of cardiovascular outcomes in type 2 diabetic patients. Diabetes Care.

[12] Schisano B, Tripathi G, McGee K, McTernan PG, Ceriello A (2011). Glucose oscillations, more than constant high glucose, induce p53 activation and a metabolic memory in human endothelial cells. Diabetologia.

[13] Habte-Asres HH, Murrells T, Nitsch D, Wheeler DC, Forbes A (2022). Glycaemic variability and progression of chronic kidney disease in people with diabetes and comorbid kidney disease: retrospective cohort study. Diabetes Res Clin Pract.

[14] Quagliaro L, Piconi L, Assaloni R, Martinelli L, Motz E, Ceriello A (2003). Intermittent high glucose enhances apoptosis related to oxidative stress in human umbilical vein endothelial cells: the role of protein kinase C and NAD(P)H-oxidase activation. Diabetes.

[15] Monnier L, Mas E, Ginet C, Michel F, Villon L, Cristol JP, Colette C (2006). Activation of oxidative stress by acute glucose fluctuations compared with sustained chronic hyperglycemia in patients with type 2 diabetes. JAMA.

[16] Inaba Y, Tsutsumi C, Haseda F, Fujisawa R, Mitsui S, Sano H, Terasaki J, Hanafusa T, Imagawa A (2018). Impact of glycemic variability on the levels of endothelial progenitor cells in patients with type 1 diabetes. Diabetol Int.

[17] Klimontov VV, Saik OV, Korbut AI (2021). Glucose variability: how does it work?. Int J Mol Sci.

[18] Ricks J, Molnar MZ, Kovesdy CP, Shah A, Nissenson AR, Williams M, Kalantar-Zadeh K (2012). Glycemic control and cardiovascular mortality in hemodialysis patients with diabetes: a 6-year cohort study. Diabetes.

[19] Jin YP, Su XF, Yin GP, Xu XH, Lou JZ, Chen JJ, Zhou Y, Lan J, Jiang B, Li Z (2015). Blood glucose fluctuations in hemodialysis patients with end stage diabetic nephropathy. J Diabetes Complications.

[20] Danne T, Nimri R, Battelino T, Bergenstal RM, Close KL, DeVries JH, Garg S, Heinemann L, Hirsch I, Amiel SA (2017). International consensus on use of continuous glucose monitoring. Diabetes Care.

[21] Buschur EO, Campbell K, Pyle L, Garcetti R, Joshee P, Demmitt JK, Snell-Bergeon JK, Polsky S (2021). Exploratory analysis of glycemic control and variability over gestation among pregnant women with type 1 diabetes. Diabetes Technol Ther.

[22] Perea V, Gimenez M, Amor AJ, Bellart J, Conget I, Vinagre I (2019). Prepregnancy care in women with type 1 diabetes improves HbA1c and glucose variability without worsening hypoglycaemia time and awareness: glycaemic variability during prepregnancy care. Diabetes Res Clin Pract.

[23] Foreman YD, Brouwers M, van der Kallen CJH, Pagen DME, van Greevenbroek MMJ, Henry RMA, Koster A, Wesselius A, Schaper NC, Stehouwer CDA (2020). Glucose variability assessed with continuous glucose monitoring: reliability, reference values, and correlations with established glycemic indices-the maastricht study. Diabetes Technol Ther.

[24] Meier JJ, Baller B, Menge BA, Gallwitz B, Schmidt WE, Nauck MA (2009). Excess glycaemic excursions after an oral glucose tolerance test compared with a mixed meal challenge and self-measured home glucose profiles: is the OGTT a valid predictor of postprandial hyperglycaemia and vice versa?. Diabetes Obes Metab.

[25] Feig DS, Shah BR, Lipscombe LL, Wu CF, Ray JG, Lowe J, Hwee J, Booth GL (2013). Preeclampsia as a risk factor for diabetes: a population-based cohort study. PLoS Med.

[26] Li-Zhen L, Yun X, Xiao-Dong Z, Shu-Bin H, Zi-Lian W, Adrian Sandra D, Bin L (2019). Evaluation of guidelines on the screening and diagnosis of gestational diabetes mellitus: systematic review. BMJ Open.

[27] Force USPST, Davidson KW, Barry MJ, Mangione CM, Cabana M, Caughey AB, Davis EM, Donahue KE, Doubeni CA, Kubik M (2021). Screening for gestational diabetes: US preventive services task force recommendation statement. JAMA..

[28] Minschart C, Beunen K, Benhalima K (2021). An update on screening strategies for gestational diabetes mellitus: a narrative review. Diabetes Metab Syndr Obes.

[29] Sweeting AN, Ross GP, Hyett J, Molyneaux L, Constantino M, Harding AJ, Wong J (2016). Gestational diabetes mellitus in early pregnancy: evidence for poor pregnancy outcomes despite treatment. Diabetes Care.

[30] Wexler DJ, Powe CE, Barbour LA, Buchanan T, Coustan DR, Corcoy R, Damm P, Dunne F, Feig DS, Ferrara A (2018). Research gaps in gestational diabetes mellitus: executive summary of a national institute of diabetes and digestive and kidney diseases workshop. Obstet Gynecol.

[31] Itakura A, Shoji S, Shigeru A, Kotaro F, Junichi H, Hironobu H, Kamei Y, Eiji K, Shintaro M, Ryu M, et al. Guidelines for obstetrical practice in Japan: Japan Society of Obstetrics and Gynecology and Japan Association of Obstetricians and Gynecologists 2020 edition. J Obstet Gynaecol Res. 2023;49(1):5–53. 10.1111/jog.15438 10.1111/jog.15438 36251613

[32] Kyozuka H, Yasuda S, Murata T, Fukuda T, Yamaguchi A, Kanno A, Sato A, Ogata Y, Hosoya M, Yasumura S (2021). Adverse obstetric outcomes in early-diagnosed gestational diabetes mellitus: The Japan Environment and Children's study. J Diabetes Investig.

[33] Brown MA, Magee LA, Kenny LC, Karumanchi SA, McCarthy FP, Saito S, Hall DR, Warren CE, Adoyi G, Ishaku S (2018). Hypertensive disorders of pregnancy: ISSHP classification, diagnosis, and management recommendations for international practice. Hypertension.

[34] Itabashi K, Miura F, Uehara R, Nakamura Y (2014). New Japanese neonatal anthropometric charts for gestational age at birth. Pediatr Int.

[35] Kapur A, McIntyre HD, Hod M (2019). Type 2 diabetes in pregnancy. Endocrinol Metab Clin.

[36] Shou C, Wei Y-M, Wang C, Yang H-X (2019). Updates in long-term maternal and fetal adverse effects of gestational diabetes mellitus. Maternal-Fetal Med.

[37] Belkacemi L, Lash GE, Macdonald-Goodfellow SK, Caldwell JD, Graham CH (2005). Inhibition of human trophoblast invasiveness by high glucose concentrations. J Clin Endocrinol Metab.

[38] Tao J, Xia LZ, Chen JJ, Zeng JF, Meng J, Wu S, Wang Z (2020). High glucose condition inhibits trophoblast proliferation, migration and invasion by downregulating placental growth factor expression. J Obstet Gynaecol Res.

[39] Majali-Martinez A, Weiss-Fuchs U, Miedl H, Forstner D, Bandres-Meriz J, Hoch D, Djelmis J, Ivanisevic M, Hiden U, Gauster M (2021). Type 1 diabetes mellitus and the first trimester placenta: hyperglycemia-induced effects on trophoblast proliferation, cell cycle regulators, and invasion. Int J Mol Sci.

[40] Vega M, Mauro M, Williams Z (2019). Direct toxicity of insulin on the human placenta and protection by metformin. Fertil Steril.

[41] Risso A, Mercuri F, Quagliaro L, Damante G, Ceriello A (2001). Intermittent high glucose enhances apoptosis in human umbilical vein endothelial cells in culture. Am J Physiol Endocrinol Metab.

[42] Yogev, Chen, Hod, Coustan, Oats, McIntyre, Metzger, Lowe, Dyer, Dooley (2010). Hyperglycemia and adverse pregnancy outcome (HAPO) study: preeclampsia. Am J Obstet Gynecol.

[43] Zhou Z, Chen G, Fan D, Rao J, Li P, Wu S, Lin D, Ma H, Ye S, Zhang H (2020). Size and shape of associations of OGTT as well as mediating effects on adverse pregnancy outcomes among women with gestational diabetes mellitus: population-based study from Southern Han Chinese. Front Endocrinol (Lausanne).

[44] Yogev Y, Xenakis EM, Langer O (2004). The association between preeclampsia and the severity of gestational diabetes: the impact of glycemic control. Am J Obstet Gynecol.

[45] Sermer M, Naylor CD, Gare DJ, Kenshole AB, Ritchie JW, Farine D, Cohen HR, McArthur K, Holzapfel S, Biringer A (1995). Impact of increasing carbohydrate intolerance on maternal-fetal outcomes in 3637 women without gestational diabetes. The Toronto Tri-Hospital Gestational Diabetes Project. Am J Obstet Gynecol.

[46] Tiselko AV, Kapustin RV, Milyutina YP, Borovik NV, Abashova EI, Yarmolinskaya MI (2022). Glucose variability as the risk factor of preeclampsia in pregnant patients with type 1 diabetes mellitus. J Matern Fetal Neonatal Med.

[47] Rolnik DL, Nicolaides KH, Poon LC (2022). Prevention of preeclampsia with aspirin. Am J Obstet Gynecol.

[48] Chung ST, Ha J, Onuzuruike AU, Kasturi K, Galvan-De La Cruz M, Bingham BA, Baker RL, Utumatwishima JN, Mabundo LS, Ricks M (2017). Time to glucose peak during an oral glucose tolerance test identifies prediabetes risk. Clin Endocrinol (Oxf).

[49] Cree-Green M, Xie D, Rahat H, Garcia-Reyes Y, Bergman BC, Scherzinger A, Diniz Behn C, Chan CL, Kelsey MM, Pyle L (2018). Oral glucose tolerance test glucose peak time is most predictive of prediabetes and hepatic steatosis in obese girls. J Endocr Soc.

[50] Wang C, Wei Y, Yang Y, Su R, Song G, Kong L, Yang H (2021). Evaluation of the value of fasting plasma glucose in the first trimester for the prediction of adverse pregnancy outcomes. Diabetes Res Clin Pract.

[51] Stekkinger E, Zandstra M, Peeters LLH, Spaanderman MEA (2009). Early-onset preeclampsia and the prevalence of postpartum metabolic syndrome. Obstet Gynecol.

