# Supplementary figures and images for: Evaluating glucose variability through OGTT in early pregnancy and its association with hypertensive disorders of pregnancy in non-diabetic pregnancies: a large-scale multi-center retrospective study
Source: Diabetol Metab Syndr. 2023 Jun 9;15:123. doi: 10.1186/s13098-023-01103-z (PMC10251636; doi:10.1186/s13098-023-01103-z)

# A

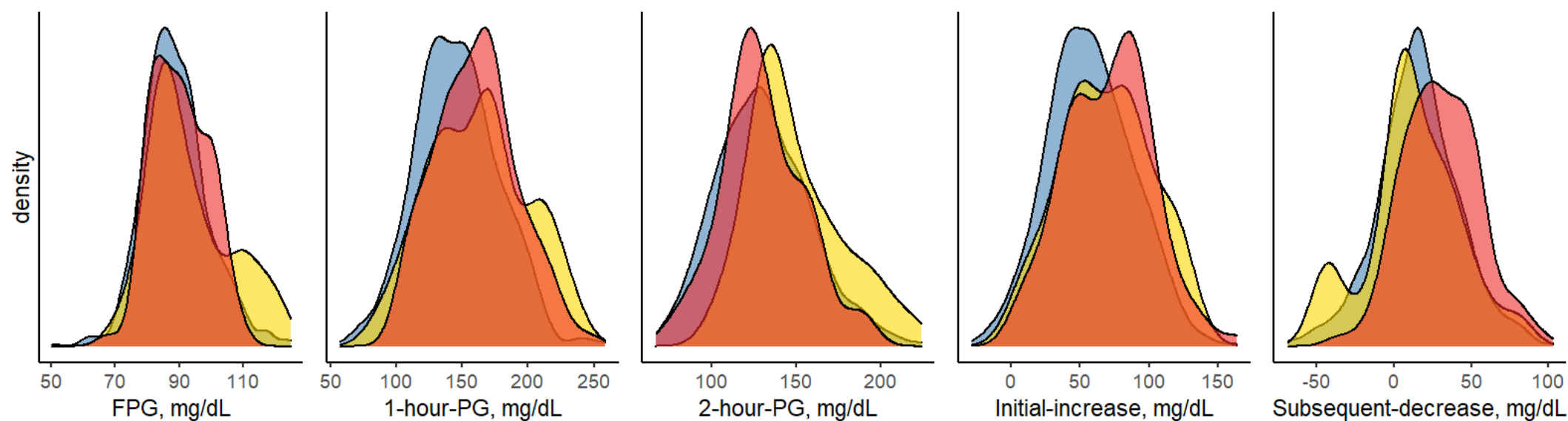

# B

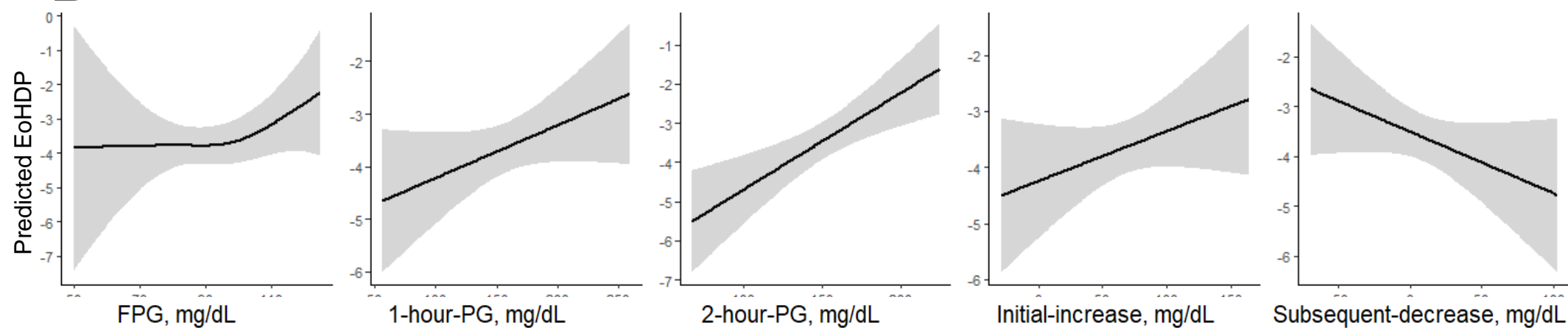

# C

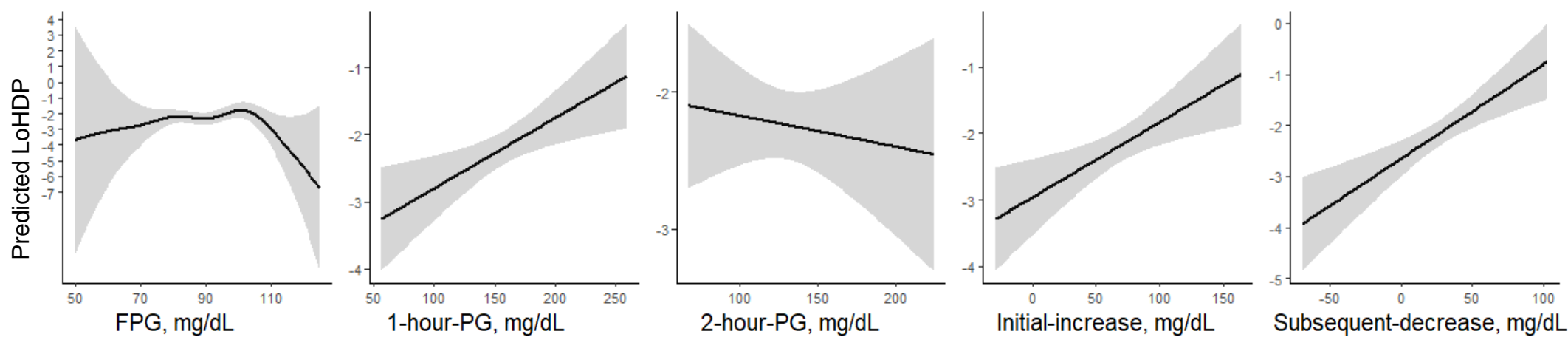

Supplement: Supplementary file 1 — Additional file 1: Figure S1. Association between 75 g-OGTT parameters and HDP subtypes (n = 802). A The X-axis of the density plot represents the 75 g-OGTT parameters. Blue, yellow, and red parts represent the non-HDP, EoHDP, and LoHDP group, respectively. B The fitted value (solid lines) and 95% CI (shaded areas) of EoHDP for each 75 g-OGTT parameters were calculated by a generalized additive model using maternal age, pre-pregnant BMI, and primiparity as covariables. C The fitted value (solid lines) and 95% CI (shaded areas) of LoHDP for each 75 g-OGTT parameter were calculated by a generalized additive model using maternal age, pre-pregnant BMI, and primiparity as covariables. FPG, fasting plasma glucose level; PG, plasma glucose level; HDP, hypertensive disorders of pregnancy; EoHDP, early-onset HDP; LoHDP, late-onset HDP; BMI, pre-pregnant body mass index. [file 13098_2023_1103_MOESM1_ESM.pdf]
